# Measuring social capital in a known disadvantaged urban community – health policy implications

**DOI:** 10.1186/1743-8462-3-2

**Published:** 2006-04-21

**Authors:** Anne W Taylor, Carmel Williams, Eleonora Dal Grande, Michelle Herriot

**Affiliations:** 1 Population Research and Outcome Studies, SA Department of Health, PO Box 287, Rundle Mall, Adelaide, 5000, Australia; 2 Health Promotion South Australia, SA Department of Health, PO Box 287, Rundle Mall, Adelaide, 5000, Australia

## Abstract

**Background:**

To assess the social capital profile of a known disadvantaged area a large cross-sectional survey was undertaken. The social capital profile of this area was compared to data from the whole of the state. The overall health status of the disadvantaged area was assessed in relation to a wide variety of social capital related variables. Univariate and multivariate analysis were undertaken.

**Results:**

In the univariate analysis many statistically significant differences were found between the respondents in the disadvantaged area and the state estimates including overall health status, perceived attributes of the neighbourhood, levels of trust, community involvement and social activities. In the multivariate analysis very few variables were found to be statistically significantly associated with poorer health status. The variables that jointly predicted poorer health status in the disadvantaged area were older age, lower income, low sport participation, non-seeking help from neighbours and non-attendance at public meetings.

**Conclusion:**

Measuring social capital on a population level is complex and the use of epidemiologically-based population surveys does not produce overly valuable results. The inter-relational/dependence dichotomy of social capital is not yet fully understood making meaningful measurement in the broader population extremely difficult and hence is of questionable value for policy decision making.

## Background

The notion of social capital and its relationship to the health of communities and individuals has sparked considerable debate within the health and health policy academic literature in recent years. [1-5] This debate has centred around how social capital is defined and how it should be measured. There appears to be general agreement amongst researchers that social capital has the ability for actors to secure benefits by virtue of membership in social networks and other social structures. [6] There is also growing agreement that it is a multi-dimensional concept, encompassing participation in social networks, trust, social norms, political participation and reciprocity and that these concepts are the property of groups, not individuals although individuals may draw benefits from it. [7]

The literature does not provide a consistent theoretical definition of social capital – What it is, who benefits from it and how can it be measured? This lack of clarity can be partly explained by the variety of disciplines that have examined the concept. For example, the work of Bourdieu, a French sociologist, defined social capital "as the aggregate of the potential resources which are linked to possessions of a durable network of more or less institutional relationships of mutual acquaintance and recognition". [6] In this definition Bourdieu places emphasis on the economic nature of social capital and how it can transfer resources and power within social groups or from one social group to another. [2,6] Coleman, an educational sociologist, defined social capital by its function and its role in the creation of human capital. [6] Robert Putnam, a political scientist, introduced the concept that social capital is a feature of communities and not individuals, he describes social capital as "the features of social organisations, such as network, norms and trust that facilitate action and cooperation for mutual benefit". [6] Specifically he describes social capital as the quantity and quality of social relationships – informal and formal connections as well as norms of reciprocity and trust that exist in a place or a community. [8-10] Finally Portes in his review of social capital argued that social capital has three main functions: a source of social control; a source of family support; and a source of benefits through extrafamilial networks. [6]

It is somewhat unclear how the concept of social capital contributes to improving the health of disadvantaged communities at a population level. Although, aspects of the above definitions can be said to have an impact on the social determinants of health, education, social support and cohesion, work (and unemployment) are seen as the underlying factors that can create or harm the health of individuals and populations. Social capital can be thought to play a role protecting and/or enhancing the health of disadvantaged communities through strong social networks and norms. It is now recognised that social capital can have both positive and negative effects. For instance one group with strong bonding social capital (relationships with people like you), may exclude and discriminate against other groups.

There is a plethora of ways to measure social capital and consensus on measurement indicators is still lacking. [1,8,11-14] It is recognised that measurement of social capital based on Coleman's definition as used in this analysis, (in which social capital is created by individuals who trust, display reciprocity and involve themselves in their community), are more easily measured quantitatively. [15] This is based on the presumption that social capital can be treated somewhat similar to other risk factors for ill health and can therefore be reliably measured via population surveys. [16]

In South Australia in 2001 the metropolitan suburbs of Kilburn (below the 10^th ^percentile of disadvantage areas in South Australia) and Blair Athol (in the 10^th ^percentile of disadvantage areas in South Australia) [17], were recognised as communities of disadvantage and were selected to be involved in a community capacity building project as a new initiative from the Department of Human Services (now the Department of Health (DH)). The baseline data from that project formed the basis for this analysis.

While many studies have assessed various forms of social capital in disadvantaged communities, the research presented in this paper was able to compare the known disadvantaged area, prior to any intervention in the disadvantaged area, with the rest of the metropolitan area and the rest of the state using a series of large-scale population surveys of randomly selected adults and a variety of measures of social capital. The aim of this analysis is to assess the relationship of these concepts with health status. In addition, this analysis aims to add to the understanding of social capital, and to contribute to this complex debate about the role of social capital in creating healthy communities.

## Method

Data from three major population surveys were used. Firstly, households in the suburbs of Kilburn and Blair Athol (KBA) in Adelaide, South Australia (SA) that had a listing in the Electronic White Pages telephone directory were randomly selected for inclusion in the sampling frame. All selected households were sent an approach letter and interviews were conducted in the respondents' homes. Within the household, a person, aged 18 years and over, who was last to have a birthday was selected to participate in the survey. There was no replacement for refusal or non-response. Up to 10 call-backs were made to each selected household for an interview. The questions were pilot tested (n = 50) and the interviews were conducted in August and September 2001. The data were weighted by age, gender, household size and geographical area to the KBA population using Australian Bureau of Statistics (ABS) residential population references. [18] In the KBA face-to-face survey 802 randomly selected adults were interviewed with a response rate of 72.4%.

To compare residents living in KBA with other populations, key questions were selected from the KBA Community Survey and included two other population state-wide surveys – the 2001 SA Health Omnibus Survey (HOS) and the 2002 March Health Monitor (HM) survey. The HOS is an annual representative health survey of people aged 15 years and over conducted each year (October/November) in metropolitan Adelaide and country SA since 1990. [19] The survey sample is a clustered, multi-stage, systematic, self-weighting area sample and is based on ABS Collector Districts and motels, hotels, hostels, hospitals and other institutions are excluded. [20] At each selected household the adult with the last birthday was selected for interview. There were no replacements for non-participants. The data were weighted by age, gender, household size and geographical area to the 2000 estimated residential population. [16] Only responses from those aged 18 years and over were included in the analyses. The methodology of HOS has been extensively reviewed. [21-24] The HOS statewide survey achieved a response rate of 71.3% with 3037 completed interviews conducted.

The HM is a representative health survey, utilising CATI (computer assisted telephone interviewing) technology, conducted three times a year (March, July, November), on people aged 18 years and over in SA. [25] Households with a telephone connected and listed in the South Australian Electronic White Pages were randomly selected to take part in the survey. An introductory letter was sent to potential respondents informing them of the survey. At each selected household the adult with the last birthday was selected for interview. There was no replacements of non-participants. The data were weighted by age, gender, household size and geographical area to the 2000 estimated residential population. [16] This comparable state-wide telephone survey had 2005 interviews with a response rate of 67.7%.

The questions included in the survey were based on two key Australian surveys; the first undertaken in New South Wales [26] in 1995 and the second in the Western Suburbs of South Australia in the late 1990's. [27,28] These included demographic characteristics, and dimensions of social capital which were grouped into five categories: type of neighbourhood, participation in community and civic activities, social participation, neighbourhood perceptions and relationships, and levels of trust and reciprocity.

The overall health question used was the first question of the MOS Short Form 36 (SF36) [29] that is commonly referred to as the SF1 or self-reported health. This question refers to physical and mental health, as assessed by individuals, according to their own values, and has been found to be a strong indicator of future health care use and mortality. [30] There are five response categories that respondents can rate their overall health status: excellent, very good, good, fair or poor.

The data was analysed using SPSS version 11.0 and Epi Info 6.0. The conventional 5% level was used to determine statistical significance. Univariate analyses used χ^2 ^tests to assess the difference between health status and demographic and social capital related variables; and to compare the people living in the KBA area compared to South Australian and Metropolitan Adelaide residents on social capital related variables. Adjusted standardized residuals were obtained using the methods of Haberman [31] and were used to test deviations from expected values separately in each cell. Bonferroni corrections were applied for multiple testing.

To determine the inclusion of independent variables in logistic regression modelling, a 'p' value of 0.25 was chosen as the critical value for statistical significance at the univariate level. [32] Backward stepwise logistic regression was used and the log likelihood was used to determine which variables were removed from the model. Variables to be entered into the logistic regression were also refitted in STATA v5.0, in order to check for multi-collinearity.

## Results

When comparing the KBA to the state-wide HOS survey, KBA respondents were statistically significantly more likely to be male (52.6% for KBA compared to 48.6% for SA), to be aged 55 years and over (37.5% for KBA compared to 31.4% for SA), to be living alone (31.3% for KBA compared to 12.2% for SA) or be an adult living with children (13.2% for KBA compared to 3.6% for SA), to have had no formal schooling or to have their highest educational attainment to be some or all primary schooling (12.5% for KBA compared to 6.8% for SA), to have government pension or other government payment as their main source of income (51.3% for KBA compared to 24.7% for SA), and to have an annual household income of $20,000 or less (49.0% for KBA compared to 23.6% for SA). There was little difference in length of time respondents had lived in the area with 51.6% of KBA respondents having lived in the area for 10 or more years (compared to 54.1% for SA as a whole, 52.1% for metropolitan Adelaide).

Overall 73.7% of KBA respondents reported excellent, very good or good general health compared to 79.5% of the state as a whole (χ^2 ^= 12.56, p < 0.001), and 79.2% of adults living in metropolitan Adelaide (χ^2 ^= 10.00, p = 0.002).

Table 1 shows the comparison between KBA respondents and the general South Australian and metropolitan Adelaide population on questions assessing perception of the neighbourhood with KBA respondents statistically significantly less likely to report positive attributes of their neighbourhood on all four measures. When variables assessing levels of trust (Table 1) were compared KBA respondents were statistically significantly more likely to have negative beliefs than the comparison groups for both measures. Table 1 highlights the difference in reciprocity with KBA respondents less likely to have help from neighbours all or most of the time but more likely to have help from neighbours a fair bit of the time.

**Table 1:** Social capital dimensions for Kilburn/Blair Athol compared to South Australia and Metropolitan Adelaide

	**Kilburn/Blair Athol**	**South Australia**		**Metropolitan Adelaide**	
	**%**	**%**	**P value**	**%**	**P value**
**PERCEPTIONS OF NEIGHBOURHOOD**					
**A very friendly place to live?**^1^			<0.001		<0.001
1,2 (positive)	59.4	73.9	*	72.5	*
3	29.8	20.4	*	21.5	*
4,5 (negative)	8.8	5.2	*	5.2	*

**Safe place to walk around at night?**^1^			<0.001		<0.001
1,2 (positive)	21.8	52.2	*	49.8	*
3	26.8	23.4	*	25.1	
4,5 (negative)	43.1	20.0	*	20.0	*

**Does your community have a reputation for being a safe place?**^2^			<0.001		<0.001
Yes	46.2	85.8		84.4	
No	37.1	8.6		9.4	
Don't know/not sure	16.7	5.6		6.2	

**Do you feel safe in your home?**^2^			<0.001		<0.001
All of the time/Most of the time	85.5	96.5		96.0	
Some of the time/None of the time	14.5	3.4		3.8	

**LEVEL OF TRUST**					
**Most people in the area can be trusted?**^1^			<0.001		<0.001
1,2 (positive)	43.1	61.1	*	59.5	*
3	32.5	24.8	*	25.2	*
4,5 (negative)	16.2	10.3	*	10.3	*

**Do you think that in this neighbourhood people generally trust one another?**^2^			<0.001		<0.001
Yes	54.1	80.0		77.6	
No	27.1	10.3		11.7	
Don't know/not sure	18.8	9.7		10.7	

**RECIPROCITY**					
**Level of help from neighbours if and when it is needed **^1^			<0.001		<0.001
Yes – all or most of the time	59.8	68.2	*	66.0	*
Yes – a fair bit of the time, sometimes	23.1	18.8	*	19.1	*
No – none of the time	7.7	4.4	*	4.7	*
No assistance required	9.3	4.3	*	5.1	*

* denotes the category was statistically significantly different compared with KBA using adjusted standardized residuals greater than 2.0 and less than -2.0 (χ2 test)

1 – Kilburn Blair Athol survey compared to the HOS 2001, 18+ years only (face-to-face survey)

2 – Kilburn Blair Athol survey compared to HM Survey 2002, 18+ years (CATI survey)

When variables assessing civic and community involvements were analysed (Table 2) compared to the general South Australian and metropolitan Adelaide population, KBA respondents were statistically significantly more likely to be involved in four activities in the last 18 months including attending church or taking part in some other religious activities, participation in an ethnic group or their activities, involvement in the local government, involvement in co-ops (eg housing, food), and making a donation of any kind (money, clothes or blood donation), but were significantly less likely to have been on a management committee or organising committee for any local group or organisation. KBA respondents were less likely to had participated in various community actions in the last 18 months, including attending a public meeting, voting in the local council elections, and picking up other people's rubbish in a public place. However, they were more likely to have contacted an organisation to deal with a particular problem eg police or council, talked to their neighbours about a local problem. There was very little difference in undertaking volunteer work.

**Table 2:** Social capital dimensions for Kilburn/Blair Athol compared to South Australia and Metropolitan Adelaide

	**Kilburn/Blair Athol**	**South Australia**		**Metropolitan Adelaide**	
	**%**	**%**	**P value**	**%**	**P value**
**CIVIC PARTICIPATION**					
**Been involved in various activities in the past 18 months? **^1^					
A school-related group	16.1	18.0	0.20	18.5	0.14
A service club (eg Lions, CWA)	7.6	6.2	0.13	5.7	0.05
Attended church or taken part in some other religious activities	36.3	26.9	<0.001	27.4	<0.001
Participated with an ethnic group or their activities (eg Croatian, Italian club)	17.6	6.1	<0.001	6.8	<0.001
Fundraising activity	26.6	24.7	0.35	23.8	0.12
Local government	5.0	3.0	0.004	2.8	0.004
Co-ops (eg food, housing)	8.3	1.1	<0.001	1.2	<0.001
Professional group (eg business forum)	8.0	9.6	0.30	9.8	0.14
Made a donation of any kind, for example money, clothes or blood donation	80.0	71.3	<0.001	72.1	<0.001
Been on a management committee or organising committee for any local group or organisation	13.2	16.8	<0.001	14.2	0.47
None of these	9.2	18.1	<0.001	16.9	<0.001

**PARTICIPATION IN COMMUNITY ACTION IN THE LAST 18 MONTHS**					
**Participated in community action? **^2^					
Contacted an organisation to deal with a particular problem (eg police, council)	38.5	28.4	<0.001	29.3	<0.001
Attended a public meeting	9.3	11.5	0.08	8.9	<0.001
Talked to your neighbours about a local problem	45.7	38.8	<0.001	36.8	0.61
Joined together with others in the neighbourhood to address a common issue	15.2	15.8	0.37	13.9	<0.001
Voted in the local council elections	30.4	51.6	<0.001	47.3	<0.001
Made newspapers, radio and TV interested in (or aware of) a problem or an issue	6.4	7.0	0.98	6.4	0.09
Picked up other people's rubbish in a public place	58.9	62.9	0.87	62.5	0.06
None of these	3.6	14.4	<0.001	16.0	<0.001

**MEMBERSHIP**					
**Presently doing any volunteer (ie unpaid) work in the community **^1^			0.48		0.94
Yes	16.3	18.0		16.4	
No	83.7	82.0		83.6	
**Have been associated with any other local community activities in any way at all in the last 18 months **^1^			0.23		0.52
Yes	17.8	19.6		16.8	
No/Don't know/can't remember	82.2	80.3		83.0	

* denotes the category was statistically significantly different compared with KBA using adjusted standardized residuals greater than 2.0 and less than -2.0 (χ2 test)

1 – Kilburn Blair Athol survey compared to the HOS 2001, 18+ years only (face-to-face survey)

2 – Kilburn Blair Athol survey compared to the HM 2002, 18+ years (CATI survey)

Respondents were asked how often they had participated in various social activities in the past 18 months (Table 3). KBA respondents were less likely to have played sport, gone to the gym or an exercise class, a social club, a café or restaurant, a club, pub or bar, to watch a sports event, or to a party or dance. However, they were more likely to have gone to a self-help or support group.

**Table 3:** Social capital dimensions for Kilburn/Blair Athol compared to South Australia and Metropolitan Adelaide

	**Kilburn/Blair Athol**	**South Australia**		**Metropolitan Adelaide**	
	**%**	**%**	**P value**	**%**	**P value**
**SOCIAL PARTICIPATION IN THE LAST 18 MONTHS**^2^					
**Played sport**			<0.001		<0.001
Once a week or more	18.2	28.4	*	28.3	*
A few times a month/monthly	5.7	7.8	*	7.4	
A few times a year/rarely	7.7	10.8	*	11.3	*
Never	68.5	53.1	*	53.0	*
					
**Gone to the gym or an exercise class**			<0.001		<0.001
Once a week or more	9.2	18.0	*	19.8	*
A few times a month/monthly	3.7	4.0		4.5	
A few times a year/rarely	7.2	4.6	*	4.6	*
Never	79.9	73.5	*	71.1	*
					
**Gone to a Self-help or support group**			<0.001		<0.001
Once a week or more to monthly	7.0	3.4	*	3.4	*
A few times a year/rarely	3.7	3.1		2.9	
Never	89.3	93.6	*	93.7	*
					
**Gone to a social club**			0.005		0.10
Once a week or more to monthly	21.2	26.2	*	24.2	
A few times a year/rarely	9.2	10.5		10.6	
Never	69.7	63.3	*	65.2	
					
**Gone to a café or restaurant**			<0.001		<0.001
Once a week or more to monthly	51.9	69.0	*	72.4	*
A few times a year/rarely	26.5	22.9	*	21.2	*
Never	21.6	8.1	*	6.3	*
					
**Gone to a club, pub or bar**			<0.001		<0.001
Once a week or more to monthly	44.5	49.5	*	48.3	
A few times a year/rarely	20.3	27.3	*	26.2	*
Never	35.1	23.3	*	25.5	*
					
**Gone to watch a sports event**			<0.001		<0.001
Once a week or more to monthly	22.5	33.8	*	32.1	*
A few times a year/rarely	20.7	24.9	*	24.8	*
Never	56.8	41.2	*	43.1	*
					
**Gone to a party or dance**			<0.001		<0.001
Once a week or more to monthly	19.4	28.8	*	30.5	*
A few times a year/rarely	32.9	45.3	*	44.4	*
Never	47.6	25.9	*	25.2	*

* denotes the category was statistically significantly different compared with KBA using adjusted standardized residuals greater than 2.0 and less than -2.0 (χ2 test)

2 – Kilburn Blair Athol survey compared to the HM 2002, 18+ years (CATI survey)

Table 4 highlights the univariate associations between people from KBA who report fair or poor health and demographic characteristics with all associations significant at p < 0.25 reported. All of the eight demographic variables examined proved to be significant. Social capital associated variables (as reported in Tables 1 to 3) were dichotomised as appropriate and assessed for univariate association with KBA residents with fair or poor health. The results are highlighted in Table 5 with all associations at p < 0.25 listed. If variables from Tables 1 to 3 are not listed they were not found to be significant in this level of analysis.

**Table 4:** Univariate associations between people living in Kilburn and Blair Athol who reported their general health as fair or poor health, with demographic characteristics

	**Self- reported general health as Fair or Poor**			
	**n**	**%**	**OR**	**p**
**Sex**				
Male	80/379	21.0	1.00	
Female	131/421	31.1	1.69	**0.002**

**Age Group**				
18–29	19/180	10.4	1.00	
30–49	49/279	17.5	1.81	0.054
50–69	64/166	38.8	5.32	**<0.001**
70+	79/175	44.9	6.97	**<0.001**

**Country of Birth**				
English speaking	176/675	26.1	1.00	
Greek/Italian	10/17	57.3	4.04	**0.005**
Other	25/108	22.4	0.84	0.56

**Home ownership**				
Private/other	121/559	22.0	1.00	
Public	89/241	37.0	2.12	**<0.001**

**Children < 18 in household**				
Yes	45/238	18.9	1.00	
No	166/562	29.5	1.80	**0.002**

**Source income**				
Wages	32/336	9.6	1.00	
Pension/Superannuation	178/464	38.4	5.89	**<0.001**

**Education**				
Post Secondary	46/261	17.5	1.00	
Secondary only	165/539	30.6	2.07	**<0.001**

**Household income**				
More than $40,001	14/166	8.4	1.00	
$20,001–$40,000	29/164	17.6	2.32	**0.02**
Less than $20,000	149/391	38.0	6.68	**<0.001**
Don't know/Refused to say	19/78	24.1	3.50	**0.001**

Note: The weighting of the data can result in rounding discrepancies or totals not adding

OR = odds ratio

**Table 5:** Univariate associations between people living in Kilburn and Blair Athol who reported their general health fair or poor health, with variables associated with social capital

	**Self- reported general health as Fair or Poor**			
	**n**	**%**	**OR**	**p**
**Length of time lived in neighbourhood**				
Less than 10 years	72/387	18.7	1.00	
More than 10 years	138/413	33.4	2.20	**<0.001**

**Neighbourhood safe to walk around at night**				
Yes	32/174	18.7	1.00	
No	178/448	28.4	1.75	**0.01**

**Participate in school related group**				
Yes	23/129	17.6	1.00	
No	188/671	28.0	1.79	**0.02**

**Participate in fundraising activity**				
Yes	40/213	18.9	1.00	
No	170/581	29.0	1.76	**0.005**

**Participate in food housing coop**				
Yes	23/67	33.9	1.00	
No	188/733	25.6	0.63	0.11

**Participate in professional group**				
Yes	12/64	18.5	1.00	
No	199/736	27.0	1.60	0.12

**On committee/local group**				
Yes	21/105	20.1	1.00	
No	189/695	27.3	1.50	0.15

**Attended public meeting**				
Yes	14/75	18.9	1.00	
No	196/725	27.1	1.59	0.17

**Speak about disagreed issue**				
Yes	171/684	25.1	1.00	
No	39/116	33.7	1.52	0.07

**Picked up others rubbish**				
Yes	111/471	23.5	1.00	
No	100/329	30.4	1.42	**0.04**

**Played sport**				
At least a few times a month	18/153	10.3	1.00	
Less than once per month	193/629	30.7	3.74	**<0.001**

**Gone to exercise or gym**				
At least a few times a month	11/73	12.5	1.00	
Less than once per month	199/710	28.1	2.80	**0.002**

**Gone to café/restaurant**				
At least a few times a month	51/296	17.3	1.00	
Less than once per month	159/504	31.6	2.21	**<0.001**

**Gone to club, pub, bar**				
At least a few times a month	58/252	23.2	1.00	
Less than once per month	152/548	27.2	1.28	0.20

**Gone to party or dance**				
At least a few times a month	16/80	19.7	1.00	
Less than once per month	195/720	27.1	1.49	0.22

**Help from neighbours**				
At least a fair bit of the time	124/508	24.4	1.00	
Sometimes/never/none	86/292	29.6	1.30	0.13

OR = odds ratio

Table 6 highlights the five variables that remained significant in the logistic regression modelling process (χ^2^_model _= 145.94, df = 7, p < 0.001). The demographic and social capital variables that best jointly predict fair or poor health for people living in the KBA area are older age groups, those whose main source of income is from pensions, who played sport less than once per month, who rarely seek help from neighbours and who do not attend public meetings.

**Table 6:** Logistic regression analysis of the likelihood of people living in Kilburn and Blair Athol reporting their general health fair or poor health, with demographic and social characteristics

	**Self- reported general health as Fair or Poor**				
	**n**	**%**	**OR**	**(95% OR)**	**p value**
**DEMOGRAPHICS**					
					
**Age Group**					
18–29	19/180	10.4	1.00		
30–49	49/279	17.5	2.18	(1.91 – 3.99)	**0.01**
50–69	64/166	38.8	4.08	(2.22 – 7.51)	**<0.001**
70+	79/175	44.9	3.87	(2.11 – 7.08)	**<0.001**

**Source income**					
Wages	32/336	9.6	1.00		
Pension/Superannuation	178/464	38.4	3.98	(2.51 – 6.35)	**<0.001**

**SOCIAL CAPITAL**					
					
**Played sport**					
At least a few times a month	18/153	10.3	1.00		
Less than once per month	193/629	30.7	2.58	(1.48 – 4.48)	**0.001**

**Help from neighbours**					
At least a fair bit of the time	124/508	24.4	1.00		
Sometimes/Never/none required	86/292	29.6	1.45	(101 – 2.08)	**0.04**

**Attended public meeting**					
Yes	14/75	18.9	1.00		
No	196/725	27.1	1.92	(1.01 – 3.66)	**0.04**

χ^2 ^model = 145.94, df = 7, p < 0.001

Note: The weighting of the data can result in rounding discrepancies or totals not adding

OR = odds ratio

## Discussion

This study has highlighted that there are a range of social capital related variables that depict a disadvantaged community but when all variables are considered equally in multivariate analysis very few are related to those who self-report less than desirable health status, and as such, no clear policy directions are apparent. Although major studies conducted around the world over many years have shown a relationship between health and social capital, [3,33-38] the conclusion reached from this analysis is that the relationship is not that straightforward. These results reinforce Muntaner's [8] and others [1,39-42] views that the relationship between health and social capital is ambiguous. As such, these results add further to the debate between the relationship between self-rated health status and various measures of social capital as well as adding to the theoretical and empirical literature.

Weaknesses in the study are acknowledged. It has been argued that population surveys should not be used to measure social capital [11,43] until more appropriate concepts are determined. This research has shown that either survey data are inadequate to explain what social capital in a community is about or conversely social capital is still ill defined to be measured in this way. In addition, the use of two different methodologies (CATI and face-to-face) could produce some of the statistically significant differences although other studies have shown very little differences in most estimates using these methodologies. [44,45] The study could have also been strengthened by the inclusion of more than one health variable to be used for comparison purposes although many studies have used this general health question as their comparison question [46,47] and there was a very significant difference between KBA respondents and the other comparison groups reinforcing the use of this single measure.

Notwithstanding there are many strengths to the study. The somewhat unexpected results when measuring aspects of social capital at community level, add considerably to the endeavour to increase understanding of the concept. Although, as yet, there is no proof that interventions build social capital or reduce health inequalities the DH is committed to this project and adding to research that aims to demonstrate health improvements as a result of increased social capital. The overall conclusion from this study is that measuring social capital in population surveys is not that simple and is more complex than envisaged. Studies assessing what else is happening in the community (eg crime prevention, education, poverty, unemployment, social amenities) should be commissioned. In fact the study may have added to the argument that the concept of social capital itself can be used to camouflage the negative impact that a lack of material resources (social and economic) can have on a community. KBA as described on a number of measures is a disadvantaged community, but when their responses were compared with other communities they often fared as well or better. Macro social and economic factors can have positive and negative effects on communities and individuals within those communities. Not everyone in a community will experience these factors in the same way (ie some may benefit, whilst other will not).

A further aim of this research was to add to the understanding of social capital and we had the unique opportunity to access a disadvantaged community on a wide range of indictors and then to compare this community with data from other large scale population surveys. At the univariate stage the community of interest was clearly disadvantaged in most ways it was measured with the perception of the neighbourhood, including levels of safety, obviously sub-optimal. Levels of trust were clearly very low as were most measures of civil participation although some measures (eg church attendance and participation in ethnic group activities or co-ops) were clearly higher in the disadvantaged area. Again measures of social participation at the univariate level depicted low levels of optimal health. When these variables and measures of socio-economic and demographic variables were assessed, taking all variables into equal consideration, the power and initial significant differences, were lost.

Only three of the many indicators of social capital remained in the final multivariate model (not playing sport, not receiving help from neighbours and not attending public meetings) as well as the two socio-demographic variables (older age and pension as main source of income). In many ways the interesting findings from this study are the variables not included in the final model with none of the perception of neighbourhood and levels of trust variables proving to be good predictors of less than optimal health status. In addition, of the many participation variables assessed, only one (playing sport) remained in the final model. Again the question needs to be asked if these are the variables that truly depict this missing ingredient that makes a community healthy? As argued by Muntaner "the laundry list of measurement strategies.... merely suggests that there may be a little something for everybody in social capital". [9]

This study has added to the debate but from an epidemiological and policy point of view more work has to be done. At this stage of development the concept of social capital is not well suited to population surveys – not because population surveys are incapable of undertaking the work but because the concept is complex covering a wide range of factors that can operate at the individual and geographic level. The relative importance of each of these sets of factors and their inter-relationship/dependence is yet to be fully understood, making meaningful measurement extremely difficult. In fact some authors have argued that the concept is largely a repackaging of old ideas such as community capacity, empowerment and social support. The concept of social capital needs to mature before it can be broken down into measurable bits – or perhaps it could be suggested that the intangible bit of what constitutes social capital is still to be determined. If the concept of social capital is to become more tangible then we need to respond to this challenge and answer the emerging policy question – should we be targeting people or place in measurement and interventions? Places make people and people make place!
